# Catalytically active inclusion bodies of L-lysine decarboxylase from *E. coli* for 1,5-diaminopentane production

**DOI:** 10.1038/s41598-018-24070-2

**Published:** 2018-04-11

**Authors:** Ramona Kloss, Michael H. Limberg, Ursula Mackfeld, Doris Hahn, Alexander Grünberger, Vera D. Jäger, Ulrich Krauss, Marco Oldiges, Martina Pohl

**Affiliations:** 1 Forschungszentrum Jülich GmbH, IBG-1: Biotechnology, 52425 Jülich, Germany; 2 Institute of Molecular Enzyme Technology, Heinrich-Heine-Universität Düsseldorf, Forschungszentrum Jülich, 52425 Jülich, Germany; 3 RWTH Aachen University, Institute of Biotechnology, 52074 Aachen, Germany; 4 Bioeconomy Science Center (BioSC), c/o, Forschungszentrum Jülich, 52425 Jülich, Germany; 5 Multiscale Bioengineering, Bielefeld University, Universitätsstraße 25, 33615 Bielefeld, Germany

## Abstract

Sustainable and eco-efficient alternatives for the production of platform chemicals, fuels and chemical building blocks require the development of stable, reusable and recyclable biocatalysts. Here we present a novel concept for the biocatalytic production of 1,5-diaminopentane (DAP, trivial name: cadaverine) using catalytically active inclusion bodies (CatIBs) of the constitutive L-lysine decarboxylase from *E. coli* (*Ec*LDCc-CatIBs) to process L-lysine-containing culture supernatants from *Corynebacterium glutamicum*. *Ec*LDCc-CatIBs can easily be produced in *E. coli* followed by a simple purification protocol yielding up to 43% dry CatIBs per dry cell weight. The stability and recyclability of *Ec*LDCc-CatIBs was demonstrated in (repetitive) batch experiments starting from L-lysine concentrations of 0.1 M and 1 M. *Ec*LDC-CatIBs exhibited great stability under reaction conditions with an estimated half-life of about 54 h. High conversions to DAP of 87–100% were obtained in 30–60 ml batch reactions using approx. 180–300 mg *Ec*LDCc-CatIBs, respectively. This resulted in DAP titres of up to 88.4 g l^−1^ and space-time yields of up to 660 g_DAP_ l^−1^ d^−1^ per gram dry *Ec*LDCc-CatIBs. The new process for DAP production can therefore compete with the currently best fermentative process as described in the literature.

## Introduction

An interpolation from the current state of the petrochemical industry and fossil-based energy supply to the next century predicts the exhaustion of fossil carbon sources, which can be attributed to an alarmingly rapid exploitation of limited natural deposits^1^. In particular, this applies to crude oil, due to the steadily growing demand^2,3^. Consequently, society will face a notable future price increase for fossil resources, which has already focused public interest on sustainable and eco-efficient alternatives. This has thus encouraged the biotechnology industry to develop processes for the sustainable production of platform chemicals, biofuels^4–6^, and in particular bio-based polymers^4,7^. Increasing knowledge about the prokaryotic metabolism and ongoing developments in systems engineering pave the way for the development of microbial hosts also enabling the economic production of intermediates and bulk chemicals. However, to meet economic demand, it is necessary to develop innovative concepts and improved bioprocesses.

Biotechnological workhorses such as *Escherichia coli*, *Saccharomyces cerevisiae* and *Corynebacterium glutamicum* have been engineered to produce ω-amino acids^8^, aromatic monomers^9^, diamines^10–12^, dicarboxylic acids^13–15^, diols^16^ and hydroxy acids^17^, respectively. From this broad spectrum of building blocks for biopolymer production, the linear aliphatic diamine 1,5-diaminopentane (DAP) is probably one of the most attractive options. One reason is its ability to produce fully bio-based polyamides, such as PA 5.4 and PA 5.10, based on DAP and dicarboxylic acids such as succinate^13,18^ and sebacic acid^19^, respectively. PA 5.10, in particular, exhibits material properties comparable or even superior to the widely used petroleum-based polyamide PA 6^20^.

**Figure 1:**
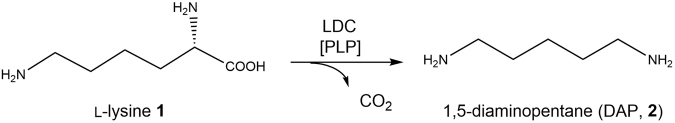
LDC-catalysed decarboxylation of L-lysine to DAP.

One option for the biotechnological production of DAP is the use of engineered, well-established L-lysine producers, especially *C. glutamicum*^10,21–24^ and *E. coli*^12,25–30^. *C. glutamicum* DAP-producer strains are usually created by the introduction of one of the L-lysine decarboxylase (LDC) genes from *E. coli* (*cadA*^21^ or *ldcC*^10^ encoding the acid-inducible enzyme CadA, and the constitutive LDCc, respectively), thus enabling the intracellular decarboxylation of the L-lysine (**1)** to DAP (**2)** (Fig. 1). Both enzymes are very similar (sequence similarity 84%)^31,32^, require the cofactor pyridoxal-5′-phosphate (PLP), and appear as decamers composed of five dimers, as was deduced from cryo-electron microscopy^33^ and X-ray crystallography^34^.

Several constraints need to be tackled for fermentative microbial production, such as the tolerance of the microbial system with respect to DAP^35^, the avoidance of by-products such as N-acetyl-1,5-diaminopentane^36^ and the management of product export^37^, which is no longer possible via the well-engineered lysine exporter LysE^38^.

Another option for DAP production is the bioconversion of L-lysine by the addition of LDC to L-lysine containing culture supernatants. Here, immobilisation of the LDC enables easy separation from the reaction medium and recycling of the biocatalyst to decrease process costs. Different concepts have been employed for the immobilisation of LDC, e.g. using whole recombinant *E. coli* cells^25,27,29,39^, immobilised recombinant *E. coli* cells in alginate beads^40,41^, as well as immobilised LDC on poly(3-hydroxybutyrate) (P(3HB) biopolymer^42^ or crosslinked enzyme aggregates (CLEAS) of LDC^43^. Generally, the bioconversions were performed in buffer and only in a few cases directly in culture supernatants of L-lysine producers^25,29^.

Catalytically active inclusion bodies (CatIBs) represent biologically produced, cell-free and carrier-free immobilisates that can easily be produced in *E. coli* cells^44–47^. They are a simple and cheap alternative to common immobilisates, which require a case-to-case optimisation of several, often expensive and laborious, steps, including chromatographic purification of the enzyme followed by covalent or non-covalent immobilisation concepts in the presence or absence of carriers^48,49^. The production of immobilised enzymes directly *in vivo* could reduce the production costs of the biocatalyst to the level of crude cell extracts^50^, since the insoluble cell fraction can be directly used for biotransformations. Furthermore, CatIB-based biotransformations are free of genetically modified organisms (GMO-free), since any remaining vital recombinant *E. coli* cells can be efficiently inactivated and separated during the production process^51^.

Active inclusion bodies can be formed either naturally by self-aggregation of the enzyme^46,52^ or by fusion to a tag containing an aggregation-prone part, e.g. cellulose binding domains^53–56^, pyruvate oxidase (PoxB) of *Paenibacillus polymyxa*^57^, the viral capsid protein VP1, the human Aβ-amyloid peptide^58^ or various self-assembling peptides^59,60^. For a detailed overview of the state of the art in this field we refer to a recent review^47^. We previously evaluated the tetramerisation domain of the cell-surface protein tetrabrachion (known as TDoT) from *Staphylothermus marinus*^61^ for its potential to induce CatIB formation. The TDoT domain has a rope-like structure forming a stable parallel tetrameric coiled coil^61^. Previous studies have demonstrated that the fusion of the TDoT domain to various enzymes of different complexity resulted in all cases in the formation of CatIBs, which implies that the TDoT-domain is a promising new fusion tag to induce the formation of active inclusion bodies^62^.

We here report on an innovative immobilisation approach using CatIBs of the constitutive L-lysine decarboxylase (*Ec*LDCc) and the application of this GMO-free approach to produce DAP in L-lysine-containing culture supernatants. In this study, we demonstrate that this approach also works for the complex PLP-dependent decameric *Ec*LDCc. This enzyme was chosen instead of the frequently used CadA mainly because of the broader pH optimum of *Ec*LDCc (pH 6.2–8) compared to CadA (pH 5.7)^63^. This property makes *Ec*LDCc advantageous for application in L-lysine-containing culture supernatants of the respective *C. glutamicum* producer strains, which exhibit pH values in the range of 6 to 8.5^64,65^.

The respective *Ec*LDCc-CatIBs were produced in *E. coli* and successfully applied in culture supernatants of a *C. glutamicum* lysine producer with lysine concentrations of up to 1 M to demonstrate the applicability of this approach on the preparative scale. Under optimised conditions, 74.7–88.4 g l^−1^ DAP was produced with 10 g l^−1^ dry *Ec*LDCc-CatIBs with a space-time yield of 296–660 g_DAP_ l^−1^ d^−1^ per gram dry *Ec*LDC-CatIBs (see Table 1).

**Table 1:** Productivity measures for the production of DAP.

Reference	Present study				Ref ^23^
Reaction mode	repetitive batch	repetitive batch	batch conversion		batch cultivation
Reaction volume [ml]	9 × 60 = 540	60	30	30	300^2^
Total reaction time [h]	69	4	24	9	50
Temperature [°C]	30	30	30	30	30
PLP [M]	0.0001	0.0001	0.0001	0.0001	—
PLP [mol]	5.4 10^−5^	5.4 10^−5^	0.3 10^−5^	0.3 10^−5^	—
L-Lys [M]	0.1	0.1	1	1	—
Total L-Lys [mol]	0.054	0.006	0.03	0.03	—
Total L-Lys [g]	7.89	0.88	4.39	4.39	—
DAP [M]	0.083^1^	0.098	0.87	0.73	0.86
DAP [g l^−1^]	8.47	9.99	88.4	74.7	88
Total DAP [g]	4.57^1^	0.599	2.65	2.24	n.d.^2^
Total DAP [mol]	0.0447	0.0059	0.026	0.0219	n.d.^2^
*Ec*LDCc-CatIB [mg ml^−1^]	3	3	10	10	—
Total amount of dry biocatalyst [mg]	180 (CatIBs)	180 (CatIBs)	300 (CatIBs)	300 (CatIBs)	n.d.^2^ (*C. glutamicum)*
Respective WCW of *E. coli* for biocatalyst production [g]^3^	1.4	1.4	2.3	2.3	—
Enzymatic productivity g_DAP_/g_biocatalyst_	25	3.33	8.8	7.5	n.d.^2^
STY [g l^−1^ d^−1^]	2.94	—	89	198	52.8
STY [g l^−1^ d^−1^] per g dry CatIBs	16	—	296	660	—
STY [g l^−1^ d^−1^] per g_*E*_. _*coli*(WCW)_	2.1	—	38	86	—
TTN [mol_DAP_ mol_PLP_^−1^]	547–994	978	8,667	7,300	—

^1^Calculated over all batches.

^2^300 ml start volume (no i.nformation concerning final volume available).

^3^Refers to previous line.

## Results and Discussion

### Production of *Ec*LDCc-CatIBs

The gene encoding the constitutive *Ec*LDCc was introduced into a pET28 vector already containing the gene sequences encoding the coiled-coil domain TDoT and an additional 3xGGGS linker region as described elsewhere^62^. Based on the quaternary structure of *Ec*LDCc, the TDoT domain was fused to the C-terminus, since the N-terminus is located within the protein structure^33^.

**Figure 2:**
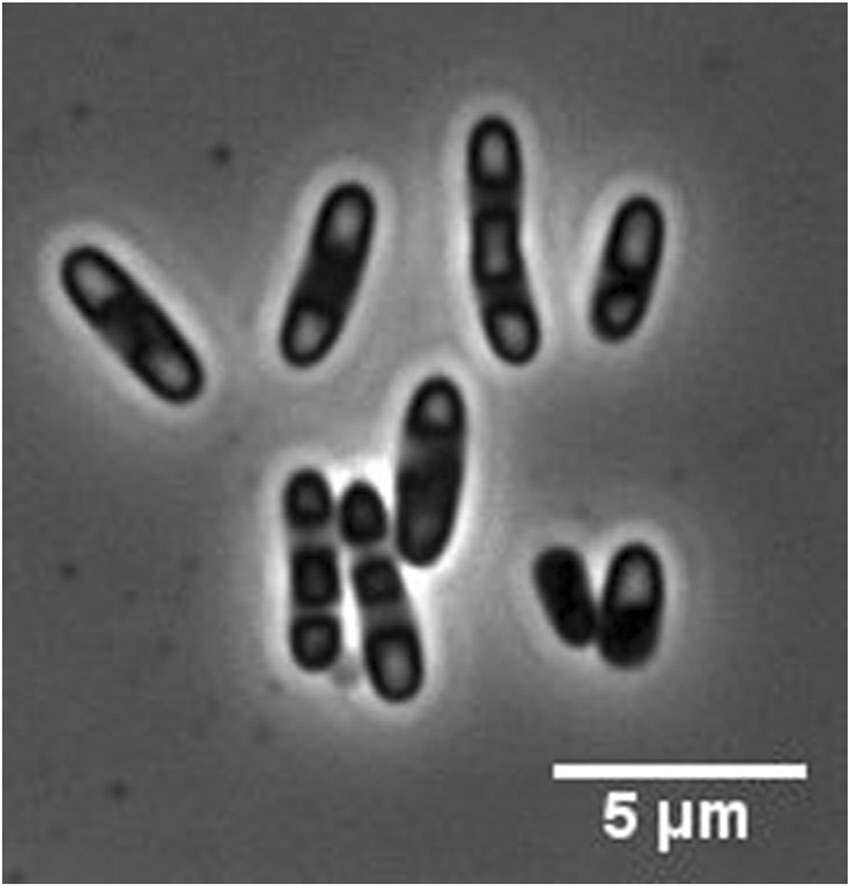
Live cell images of *E. coli* BL21(DE3) cells containing *Ec*LDCc-CatIBs. For details see Supplementary “Live cell imaging.”

*Ec*LDCc-CatIBs could be easily produced in *E. coli* BL21(DE3) using an auto-induction medium (see Sect. 5.3). The formation of *Ec*LDCc-CatIBs in the respective recombinant *E. coli* cells was demonstrated with inverted epifluorescence microscopy^66,67^ showing the CatIBs as bright spots at the cell poles (Fig. 2), which is typical of the deposition of recombinant proteins as inclusion bodies in *E. coli*^68^.

**Figure 3:**
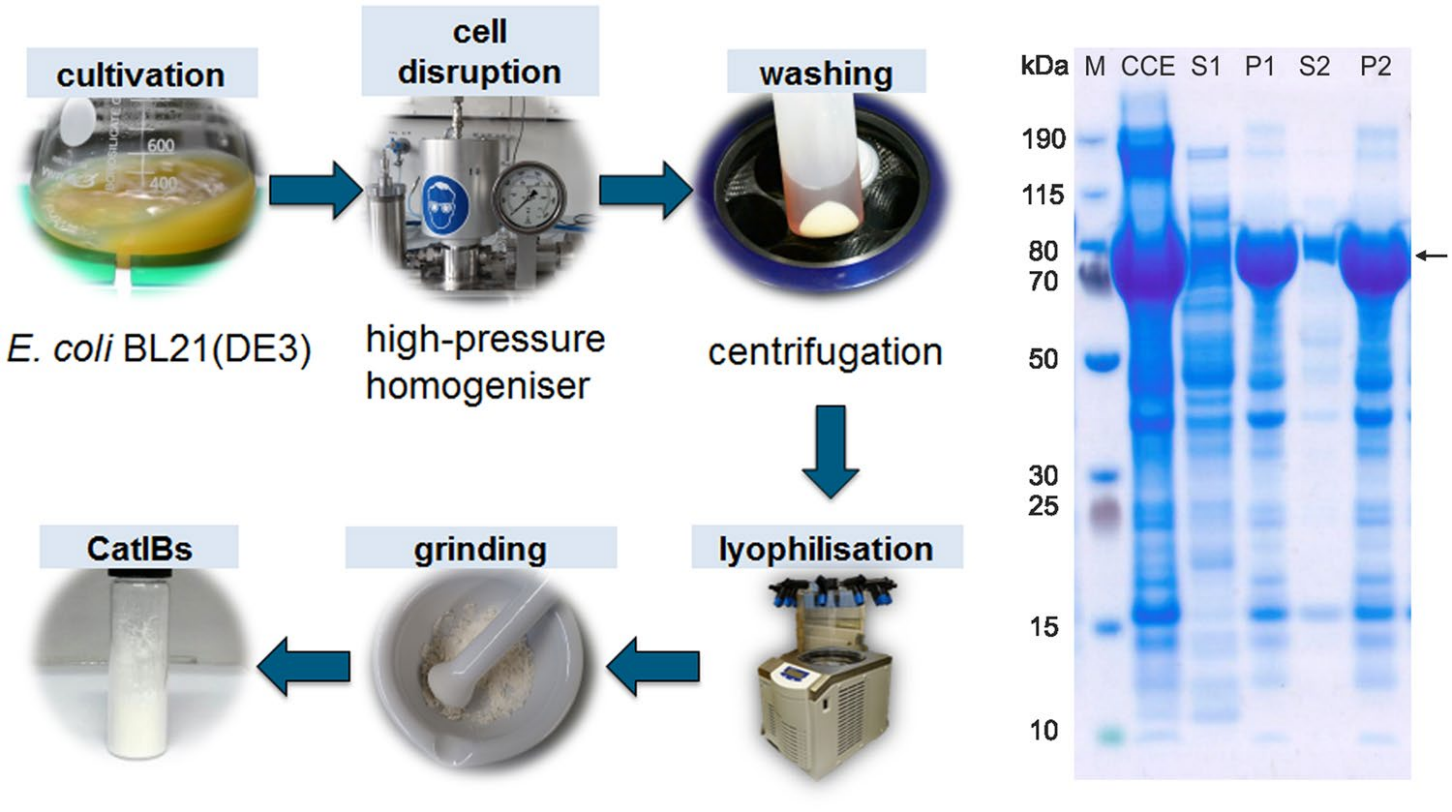
Left: Production and purification of *Ec*LDCc-CatIBs produced in *E. coli* BL21(DE3). Right: SDS-PAGE analysis of the *Ec*LDCc-CatIB preparation (calculated molecular weight: 87.8 kDa, arrow); CCE = crude cell extract, which was centrifuged to separate the supernatant (S1) from the pellet (P1). The pellet P1 was washed once with MilliQ water by resuspension and subsequent centrifugation, resulting in S2 and P2; the protein concentration was measured using the Bradford assay (see Methods). For SDS-PAGE, samples were diluted with water to a protein concentration of 1 mg ml^−1^ by the following dilution factors: 4 for CCE, 2 for S1 and P1, 4.5 for P2; 1 for S2; M = Marker. For details see Methods.

A previously developed protocol^62^ was further optimised for the purification of *Ec*LDCc-CatIBs (see Fig. 3, left). After two washing steps with water followed by centrifugation, the pellet containing the CatIBs and some cell membrane fractions was lyophilised, yielding 130 ± 37 mg dry CatIBs per gram of wet cells (approx. 13% of the wet cell weight, corresponding to 43% dry CatIBs based on dry cell weight). The protein content of the pellet was about 68%, which is comparable to previous results obtained with other CatIB enzymes^62^. The production process was monitored by sodium dodecyl sulfate-polyacrylamide gel electrophoresis (SDS-PAGE, Fig. 3, right) showing that the *Ec*LDCc-TDoT fusion is predominantly present in the pellet. Due to the simple purification protocol (see Methods), further cellular proteins co-purified with the CatIBs were expected, as was also reported for other inclusion body formulations^69^.

The activity of the *Ec*LDCc-CatIBs was demonstrated in potassium phosphate buffer (KPi buffer) and cultivation medium (CGXII)^64^ (Supplementary Fig. S2). Additionally, the CatIBs were compared to an *E. coli* whole cell biocatalyst containing the overproduced soluble LDCc. The results demonstrate that *Ec*LDCc-CatIBs can compete with the whole cell biocatalyst (for details see Supplementary Fig. S7a,b). Subsequently, the *Ec*LDCc-CatIBs were characterised in CGXII medium and used for a case study under technical conditions in culture supernatants of a *C. glutamicum* L-lysine producer strain.

### Characterisation of *Ec*LDCc-CatIBs

#### Activity in phosphate buffer

In a first step, the *Ec*LDCc-CatIBs were characterised in KPi buffer to determine the pH optimum in the pH range of 7–9 and the minimal requirement for PLP.

**Figure 4:**
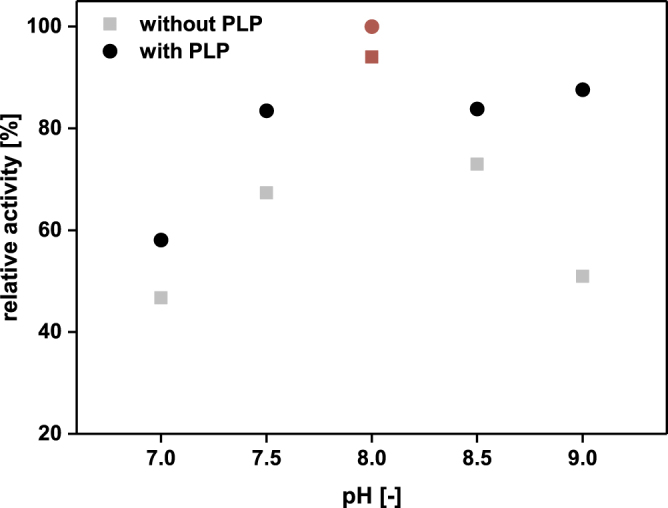
pH optimum of *Ec*LDCc-CatIBs for the decarboxylation of L-lysine in the presence (0.1 mM) and without additional PLP. For assay conditions see Methods. 100% relative activity refers to 0.34 U mg^−1^_CatIBs_, which corresponds to 0.52 U mg^−1^_protein_.

As already mentioned in the Introduction, the soluble wild-type *Ec*LDCc is active in a relatively broad pH range exhibiting maximal activity between pH 6.2 and pH 8, whereas at pH 8.8 the activity was shown to decrease to 30%^63^. As demonstrated in Fig. 4, *Ec*LDCc-CatIBs showed considerable activity between pH 7.5–9.0 with a clear activity maximum at pH 8. Furthermore, addition of the cofactor PLP was decisive in achieving optimal enzyme activity. Generally, the activity increased by 5–15% in the presence of the cofactor (Fig. 4). Strikingly, at pH 9 the positive PLP effect was approximately 35%. In additional studies, a PLP concentration of 0.05 mM was found to be sufficient for maximal activity of the *Ec*LDCc-CatIBs in buffer (see Supplementary Fig. S3). A similar positive effect of PLP on the LDC activity was recently reported for the second isoenzyme in *E. coli*, the acid-inducible CadA overproduced in recombinant *E. coli*, which was used as a whole-cell biocatalyst. In this case, full conversion of 1 M L-lysine to DAP was observed in the presence of 0.025 mM PLP, whereas without additional PLP only 20% conversion was achieved^29^.

#### Activity in CGXII minimal medium

To verify the applicability of *Ec*LDCc-CatIBs at the preparative scale, DAP production was tested in CGXII cultivation medium providing an experimental setup close to requirements on the technical scale.

First, the optimal pH was determined between pH 7.0–9.0 in fresh CGXII medium with 0.1 mM PLP and 10 mM L-lysine. *Ec*LDCc-CatIBs revealed the highest conversion between pH 8–9, showing a maximum at 8.5 (see Supplementary Fig. S4), which closely corresponds to the pH optimum in KPi buffer (Fig. 4). To ensure comparability, all subsequent experiments were performed at pH 8 providing excellent conditions for *Ec*LDCc-CatIBs in CGXII medium as well as KPi buffer.

**Figure 5:**
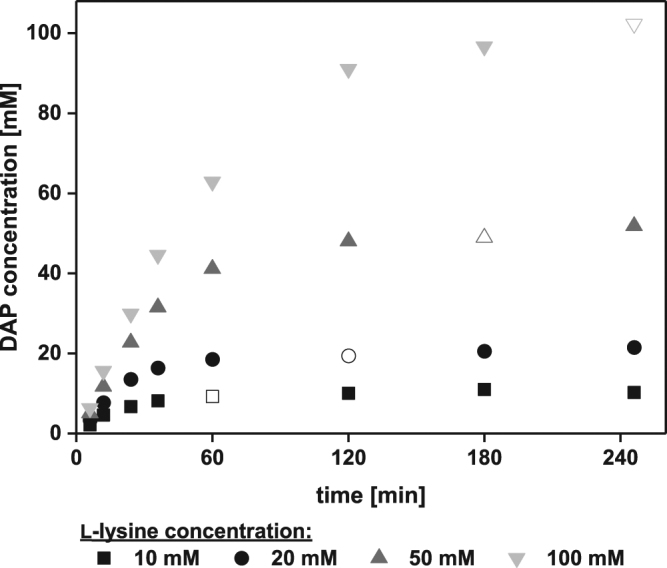
Conversion curves of the *Ec*LDCc-CatIB-catalysed decarboxylation of different L-lysine concentrations to DAP in CGXII medium. Empty symbols indicate the point in time at which full conversion was reached. 2 mg ml^−1^ lyophilised *Ec*LDCc-CatIBs. For details see Methods.

In technical processes, L-lysine concentrations of up to 120 g l^−1^ (820 mM) are expected^22^. Therefore, *Ec*LDCc-CatIBs were tested in (repetitive) batch reactions with substrate concentrations of up to 1 M L-lysine. In initial studies with 10–100 mM L-lysine, a concentration of 2 mg ml^−1^ *Ec*LDCc-CatIBs was shown to be sufficient to completely convert 100 mM L-lysine to DAP in 4 h (Fig. 5). Notably, the estimated activity increased from approx. 0.3 U mg^−1^ (10 mM L-lysine) to approx. 0.8 U mg^−1^ (100 mM L-lysine), giving rise to the conclusion that the maximum velocity (V_max_) of the CatIBs requires a L-lysine concentration of 100 mM or higher. Under the applied conditions, *Ec*LDCc-CatIBs exhibited half-maximum activity at approx. 23 mM L-lysine. This value is much higher compared to the K_M_ value of 0.84 mM published for the soluble enzyme^70^, which was determined under different reaction conditions (soluble *Ec*LDCc in 0.5 M sodium acetate buffer, pH 5.5), which makes a comparison of K_M_ values meaningless. However, the enormously increased K_M_ for the CatIBs could indicate a form of mass transport limitation of the substrate or product in the environment of the precipitated protein structure of the CatIBs. The highest enzymatic productivity of 4.9 g_DAP_ g_LDC-CatIBs_^−1^ (48 mmol_DAP_ g_LDC-CatIBs_^−1^) was achieved with 100 mM L-lysine in these first studies.

#### Application of *Ec*LDC-CatIBs for the production of DAP

Subsequently, *Ec*LDC-CatIBs were characterised in CGXII medium containing L-lysine produced by a *C. glutamicum* DM1945 strain^71^.

In the first trial, *Ec*LDCc-CatIBs were directly added to the cultivation medium to enable the simultaneous production of L-lysine followed by decarboxylation to DAP in one pot. Surprisingly, only low yields of DAP were obtained, although PLP was added to the cultivation medium. This result could be due to the degradation of PLP by photolysis or oxidation^72^ or consumption of the cofactor by *C. glutamicum*, which was earlier reported by Kind *et al*., who studied the positive effect of adding PLP to the cultivation broth of a *C. glutamicum* DAP producer strain^10^. A further reason could be the low apparent affinity of *Ec*LDCc-CatIBs to L-lysine (K_M_ approx. 23 mM) as discussed above, which results in low conversion rates at substrate concentrations <100 mM.

In order to circumvent this issue, the lysine-producing cultivation of *C. glutamicum* DM1945 was first completed in CGXII medium. The cell-free culture supernatant was further supplemented with L-lysine to 0.1 M and 0.1 mM PLP.

The initially performed determination of the stability of *Ec*LDC-CatIBs in this reaction system, as well as their continued application in repetitive batch mode, shows that *Ec*LDCc-CatIBs are fully stable for at least 24 h and can be recycled several times (for details see Supplementary Fig. S5). In repetitive batch mode, the CatIBs were reused after centrifugation and resuspension for five batch cycles, resulting in a productivity of 19.4 g_DAP_ g_LDC-CatIBs_^−1^ (190 mmol_DAP_ g_LDC-CatIBs_^−1^), which is 4 times higher compared to a single batch (Fig. 5).

**Figure 6:**
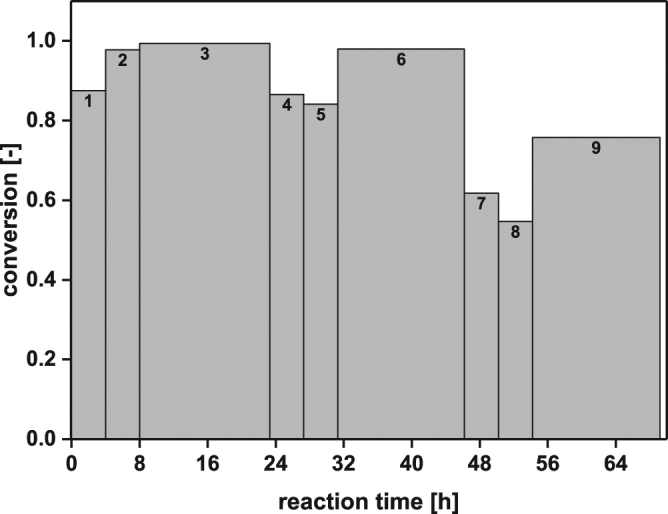
Repetitive batches for the production of DAP with *Ec*LDCc-CatIBs with pH-control. Experimental conditions: 3 mg ml^−1^ lyophilised *Ec*LDCc-CatIBs, 0.1 M L-lysine, 0.1 mM PLP in 60 ml cell-free culture supernatant (CGXII medium, pH 8). Two 4 h batches (batch 1, 2, 4, 5, 7, 8) were followed by 15 h overnight batches (3, 6, 9) on three subsequent days.

Based on these promising results, a repetitive batch on the 60 mL scale with 0.1 M L-lysine was set up in a pH-controlled environment, which was necessary since the reaction products CO_2_ and DAP shift the pH. The nine-batch cycles took either 4 or 15 hours. The results of the repetitive batch experiment revealed a constant high conversion of 84–98% during the first 46 h (Fig. 6). The first two repetitive batches (each lasting 4 h) showed almost full conversions of 90–98%. Also the 3^rd^ batch reaction (performed for 15 h) yielded full conversion. After 54 h reaction time (batch 8) the half-life of the *Ec*LDC-CatIBs has almost been achieved, since the conversion decreased to 54%. After 69 h reaction time, the 9^th^ batch (lasting 15 h) only reached 76% conversion, demonstrating that 15 h reaction time was not sufficient to compensate the progressive inactivation. By means of the repetitive batch approach, the enzymatic productivity was increased to 25 g_DAP_/g_LDC-CatIBs_ (Table 1), which is 7.5 times higher compared to a single 60 ml batch reaction (e.g. 2^nd^ batch: 3.33 g_DAP_ g_LDC-CatIBs_^−1^), yielding a final DAP concentration of 8.47 g l^−1^, a specific space-time yield (STY) of 16 g_DAP_ l^−1^ d^−1^ per gram *Ec*LDCc-CatIBs, and a total turnover number (ttn) for PLP of up to 994. This experiment showed that the *Ec*LDCc-CatIBs could be reused for several cycles for at least 69 hours under the applied conditions.

**Figure 7:**
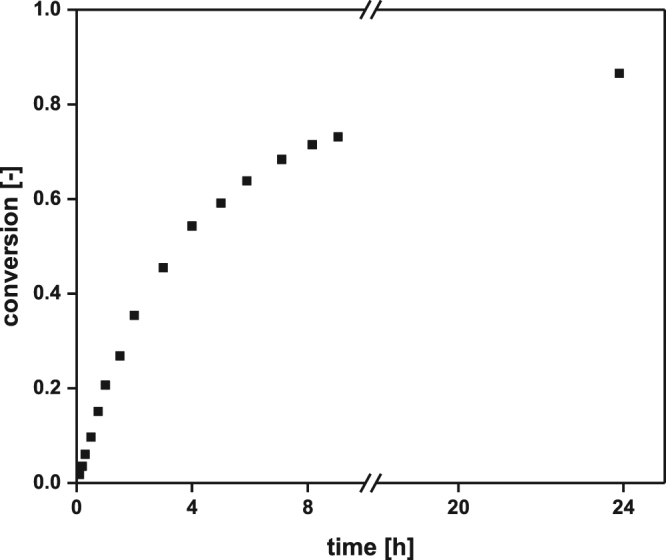
Conversion curve for the production of DAP with *Ec*LDCc-CatIBs in a 30 ml batch reactor with pH control. Experimental conditions: 10 mg ml^−1^ lyophilised *Ec*LDCc-CatIBs, 1 M L-lysine, 0.1 mM PLP, in 30 ml cell-free culture supernatant (CGXII medium, pH 8). For the dosage profile with NaOH and HCl, respectively, to keep the pH constant see Supplementary Fig. S6.

To apply the *Ec*LDCc-CatIBs under the requirements on a technical scale, where L-lysine concentrations of up to 1 M are converted to DAP^22,29^, the application was next tested in a batch reaction (30 ml) with 1 M L-lysine, which resulted in 87% conversion after approx. 24 h (Fig. 7). The specific activity of 0.75 U mg^−1^, deduced from conversions ≤10%, was comparable to the reaction velocity observed with 100 mM L-lysine (see above), which indicates that there is no substrate inhibition for *Ec*LDCc-CatIBs up to 1 M L-lysine. Although the enzymatic productivity was reduced to 30% (8.8 g_DAP_ g^−1^_CatIBs_) compared to the previous repetitive batch experiments with 100 mM L-lysine, due to the higher concentration of CatIBs, the STY was increased 180-fold to 296 g_DAP_ l^−1^ d^−1^ per gram *Ec*LDC-CatIBs. As demonstrated in Fig. 7, the reaction slowed down after a process time of 9 h and between 9 h and 24 h conversion only increased by about 10%. The analysis of the reasons for the slowdown of the reaction requires further investigation. One possibility could be inactivation of the enzyme by negative effects caused by the pH-adjustment with NaOH and HCl or due to high concentrations of DAP, which could be targeted by reaction engineering. If the high DAP concentration is the reason for deactivation of the enzyme, stopping the reaction after 9 h would be a good option (Fig. 7). This would result in a more than twofold higher specific space-time yield of 660 g_DAP_ l^−1^ d^−1^ per gram *Ec*LDC-CatIBs. Accordingly, the ttn for the cofactor PLP was increased by a factor of 10 relative to the repetitive batch with 0.1 M L-lysine (see Table 1). Consequently, a 10 times higher product concentration was reached in one third of the time (24 h) needed for the repetitive batch mode using 0.1 M L-lysine.

## Conclusions

The development of cheap, stable, reusable and recyclable biocatalysts is necessary for the prospective creation of competitive sustainable and eco-efficient production processes for platform chemicals, fuels, and polymer building blocks. One promising approach to fulfil industrial demands in terms of productivity, yield, and product titre is the application of whole-cell biotransformation^50^ e.g. using resting or metabolically active microbial cells. However, the drawbacks of this approach are productivity issues, e.g. due to undesired side reactions, negative interactions of substrates and products with the microorganism as well as difficulties in downstream processing caused by lysed cells under process conditions. Furthermore, the application of genetically modified organisms (GMO) requires conformity with the respective safety standards. In contrast to whole cells, the preparation of catalytically active inclusion bodies (CatIBs) requires only a few additional steps (cell disruption, solid/liquid separation, washing with water) and thus represents a versatile and cheap GMO-free immobilisation method.

Here we demonstrate the application of CatIBs for the production of 1,5-diaminopentane (DAP) using the constitutive decameric and PLP-dependent lysine decarboxylase from *E. coli* (*Ec*LDCc). Currently, this represents the structurally most complex enzyme in our CatIB toolbox^62^.

*Ec*LDCc-CatIBs can be produced with high yields (about 13% dry CatIBs based on the wet cell weight, equivalent to 43% dry CatIBs based on dry cell weight) at low cost comparable to crude cell extract^50^. A two-step process was applied whereby L-lysine is produced first through a *C. glutamicum* producer strain and the culture supernatant is subsequently treated with *Ec*LDCc-CatIBs to produce DAP. Maximal conversion rates were obtained with L-lysine concentrations of 0.1–1 M. As was found for soluble *Ec*LDCc^73^ and whole cell catalysts^27,29^, the addition of PLP was decisive for optimal CatIB activity. After optimisation of the reaction conditions, a study on a preparative scale demonstrated that *Ec*LDCc-CatIBs are recyclable and stable biocatalysts for DAP production directly applicable in L-lysine-containing culture supernatant. The *Ec*LDCc-CatIBs were successfully reused by simple centrifugation and resuspension steps. Starting from 1 M L-lysine, a maximal DAP concentration of 74.7–88.4 g l^−1^ and a specific STY of up to 296–660 g_DAP_ l^−1^ d^−1^ per gram *Ec*LDC-CatIBs were obtained (see Table 1). This result compares well with the currently best fermentative process using *C. glutamicum*, which also achieved a final titre of 88 g l^−1^ DAP after 50 hours of a combined batch/fed-batch fermentation, but a STY of only 52.8 g l^−1^ d^−1^ (2.2 g l^−1^ h^−1^)^23^ (see Table 1). In order to fulfil the technically relevant demands, the usage of *Ec*LDCc CatIBs in batch mode at high substrate concentrations proved to be appropriate in order to obtain high STY.

## Methods

### Materials

All chemicals were purchased from Sigma-Aldrich, Roth, KMF and Merck. Enzymes for molecular biology were purchased from Thermo Scientific.

### Cloning

See Supplementary “Cloning & sequences.”

### Protein production, cell disruption and protein purification

*Ec*LDCc-CatIBs were produced in *E. coli* BL21(DE3) as recently described elsewhere^62^. Here, a temperature of 15 °C during protein production was decisive for the formation of active *Ec*LDC-CatIBs. Cell disruption was performed with a high-pressure homogeniser (EmulsiFlex-C5, Avestin Europe GmbH, Mannheim, Germany) at 1000 bar using a cooled 10% (w/v) suspension of *E. coli* cells in cell lysis buffer (50 mM sodium phosphate, 100 mM NaCl, pH 8). To ensure thorough cell disruption, the suspension was passed three times through the high-pressure homogeniser under constant cooling. SDS-PAGE (see below) was used to analyse the distribution of the recombinant protein in the *E. coli* cells and during CatIB isolation. After cell disruption, the crude cell extract, and the soluble and insoluble protein fraction were separated by centrifugation at 15,000 × *g* for 30 min. The pellet was washed by suspension in MilliQ water in the initial volume followed by centrifugation. The obtained pellet was frozen overnight at −20 °C and a 10% (w/v) suspension in MilliQ water was prepared for lyophilisation (Christ ALPHA 1–3 LD Plus, Martin Christ Gefriertrocknungsanlagen GmbH, Osterode, Germany). In a mortar the dried pellet was ground to a fine powder, which was weighed and stored at −20 °C for further use.

### SDS-PAGE and protein assay

SDS-PAGE analysis was performed using the NuPAGE® Kit (ThermoFisher Scientific), consisting of LDS Sample Buffer (4×) and NuPAGE® Reducing Agent (10×) with a final protein content of 1 mg ml^−1^. Previously, the soluble protein concentration had been measured using the Bradford assay^74^ and bovine serum albumin as a standard. Samples were applied to a NuPAGE™ 4–12% Bis-Tris protein gel, 1.0 mm, with 15 wells together with a protein marker (PageRuler Plus Prestained Protein *ladder*, ThermoFisher Scientific). Gel electrophoresis was performed in NuPAGE® MES SDS running buffer (1×) at 200 V, 100 mA and 15 W.

The protein content of the lyophilised CatIBs was determined by absorption at 280 nm. For this purpose, a defined amount (1–2 mg) of freeze-dried CatIBs was dissolved in 6 M aqueous guanidine hydrochloride solution (1 ml) and incubated at 30 °C for 30 min under constant shaking at 1000 rpm in a thermomixer (Thermomixer comfort, Eppendorf, Germany). The absorption of the protein solution was measured at 280 nm with a spectrophotometer (Shimadzu UV-1800/UV-1600). The protein content was estimated using the molar extinction coefficient (*Ec*LDCc-CatIBs: ε = 109,210 l mol^−1^ cm^−1^) as calculated based on the amino acid composition using the ProtParam Tool (http://web.expasy.org/protparam).

### Live cell imaging

See Supplementary.

### pH optimum of *Ec*LDC-CatIBs in KPi buffer and activity assay

In order to analyse the pH optimum, a reaction with 0.5 mg ml^−1^ lyophilised *Ec*LDCc-CatIBs in a reaction tube (2 ml safe-lock tube, Eppendorf, Germany) in 1 ml KPi buffer (50 mM, pH 7.0, 7.5, 8.0, 8.5, 9.0) containing 10 mM L-lysine, 0.1 mM PLP was performed for 20 min at 30 °C and 1000 rpm in a thermomixer (Thermomixer comfort, Eppendorf, Germany). After different time intervals (5, 10 and 20 min), 20 µl of the samples was taken from one vial. The reaction was stopped by incubation at 90 °C for 2 min and subsequent centrifugation for 2 min at 15,800 × *g*. The samples were then diluted to 1:50 in KPi buffer (50 mM, pH 7.0). The specific activity was calculated based on the DAP formed within the linear range, which was determined by HPLC analysis (see below).

One unit (U) of specific activity is defined as the amount of enzyme (in mg, calculated on the basis of protein content) which catalyses the formation of 1 µmol DAP per minute from the respective L-lysine concentration under the applied reaction conditions. The formation of DAP was monitored using HPLC as described below.

### Characterisation of *Ec*LDCc-CatIBs in CGXII medium supplemented with different L-lysine concentrations

To characterise the performance of *Ec*LDCc-CatIBs for the transformation of different L-lysine concentrations from 10–100 mM, reactions of 2 mg ml^−1^ lyophilised *Ec*LDCc-CatIBs each were performed in reaction tubes (2 ml safe-lock tube, Eppendorf, Germany) in 1 ml CGXII medium^64^ adjusted to pH 8.0 and containing 10, 20, 50, and 100 mM L-lysine and 0.1 mM PLP for 4 h at 30 °C and 1000 rpm in a thermomixer (Thermomixer comfort, Eppendorf, Germany). After different time intervals (6, 12, 24, 36, 60, 120, 180, and 246 min), 20 µl samples were taken and stopped by 1:5 dilution with methanol. The reaction mixture was then 1:10 diluted in KPi buffer (50 mM, pH 8.0) and subsequently centrifuged for 2 min at 15,800 × *g*. The samples thus obtained were diluted in an appropriate manner to obtain a final DAP concentration suitable for HPLC analysis (see below) between 10 and 100 µM. The specific activity was calculated as described above.

### Bioreactor cultivation of *C. glutamicum*

See Supplementary.

### Application of *Ec*LDCc-CatIBs in a (repetitive) batch

*Ec*LDCc-CatIBs were characterised in a cell-free culture supernatant (30 or 60 ml), with an adjusted pH of 8.0 and 0.1 mM PLP. Decarboxylation reactions of 100 mM and 1 M L-lysine were performed in repetetive batch and batch experiments, respectively. For the repetitive batch experiment, 3 mg ml^−1^ *Ec*LDCc-CatIBs were used in 60 ml reaction solution. For the single batch reaction, starting from 1 M L-lysine, 10 mg ml^−1^*Ec*LDCc-CatIBs were added to 30 ml reaction solution. The experiments were performed under pH-control by dosing NaOH (2 M) and HCl (5%), respectively, using a 665 Dosimat, 632 pH meter equipped with a 614 Impulsomat from Metrohm, Germany. For the dosage profile of NaOH and HCl during the batch reaction starting from 1 M L-lysine see Supplementary Fig. S6. Reactions were performed in a doubled-walled 3-neck reactor vessel with two nozzles for the cooling supply to keep the temperature constant at 30 °C. The reaction mixture was stirred with a magnetic stirrer. For the repetitive batch approach, consecutive batch experiments were performed for either 4 h or 15 h. After a batch of 4 h or 15 h, the reaction mixture was transferred to a centrifugal beaker and centrifuged at 30,966 × *g* for 2 min. The pellet was suspended in the fresh reaction solution described above and transferred back into the doubled-walled flask reactor vessel. 20 µl samples were taken from the supernatant of the respective batch (60 ml approach) and the reaction was stopped by 1:5 dilution with methanol. The reaction mixture was then diluted 1:100 in KPi buffer (50 mM, pH 8.0). 5 µl samples were taken from the 30 ml batch experiment after different time intervals (6, 12, 18, 30, 45, 60, 90, 120, 180, 240, 300, 354, 426, 480, 543, and 1434 min) and the reaction was stopped by 1:20 dilution with methanol. The reaction mixture was then diluted (1:250) in KPi buffer (50 mM, pH 8.0). All samples were subsequently centrifuged for 2 min at 15,800 × *g*. The amount of DAP formed was determined by HPLC analysis (see below).The specific activity was calculated as described above.

### Quantification of L-lysine and DAP by HPLC

To determine the DAP concentration in cell-free and CatIB-free reaction solutions, a HPLC-system (Agilent 1100 Infinity, Agilent Technologies, Santa Clara, USA) was used, equipped with a fluorescence detector (excitation: 230 nm; emission: 460 nm) and a C18 KinetexEvo column (Phenomenex, Torrence, USA). Prior to injection, samples were diluted 1:2 (v/v) with 100 µM α-aminobutyric acid as the internal standard (Sigma-Aldrich, St. Louis; USA). Analysis of DAP and L-lysine was performed by a method for amino acid quantification^71^ including a pre-column derivatisation step at 18 °C using 5 µl *ortho*-phthaldialdehyde (OPA, Sigma-Aldrich) and 5 µl sample (6 mixing iterations). The mobile phase A was composed of 7.12 g l^−1^ Na_2_HPO_4_, 6.24 g l^−1^ NaH_2_PO_4_ and 0.8% (v/v) THF in water, and the mobile phase B contained 50% (v/v) methanol, 45% (v/v) acetonitrile, and 5% (v/v) water. For chromatographic separation, a linear gradient was applied with a flow of 1 ml min^−1^ starting with 0% B, 0–2 min 0–38% B, 2–6 min 38–42% B, 6–7 min 42–70% B, 7–9 min 70–100% B, 9–13 min 100-0% B. Approximate retention times were 8 min for α-aminobutyric acid, 10 min for L-lysine, and 11 min for DAP. In order to correct for possible effects of the analytical matrix on derivatisation efficiency, α-aminobutyric acid (Sigma-Aldrich, St. Louis; USA) was used as an internal standard. The DAP concentration was derived from the linear calibration of five reference solutions (10 µM to 100 µM), included in each measurement run (for the calibration curve see Supplementary Fig. S8).

### Data availability

The datasets generated during the current study are available from the corresponding author on reasonable request.

## Electronic supplementary material

Supplementary information
