# Supplementary material for: Catalytically active inclusion bodies of L-lysine decarboxylase from E. coli for 1,5-diaminopentane production
Source: Sci Rep. 2018 Apr 11;8:5856. doi: 10.1038/s41598-018-24070-2 (PMC5895699; doi:10.1038/s41598-018-24070-2)
Supplement: Supplementary file 1 — Supplementary information [file 41598_2018_24070_MOESM1_ESM.pdf]

Supplementary Information

Catalytically active inclusion bodies of lysine decarboxylase from *E. coli* for 1,5-diaminopentane production

Ramona Kloss[1,4+], Michael Limberg[1+], Ursula Mackfeld[1], Doris Hahn[1], Alexander Grünberger[1, 5], Vera D. Jäger[2,4], Ulrich Krauss[2,4], Marco Oldiges[1,3], and Martina Pohl[1,4]

Content

Cloning & sequences 2

Expression vector 2

Amino acid sequence of *Ec*LDCc-TDot 5

Live cell imaging 5

Bioreactor cultivation of *C. glutamicum* 5

Optimisation of the reaction system of *Ec*LDCc-CatIBs for the production of DAP from L-lysine 6

Control experiments with soluble LDCc in *E. coli* 9

HPLC calibration curves 10

References 10

Cloning & sequences

Based on the earlier described cloning strategy[1], the gene encoding *Ec*LDCc was cloned into a pET28a vector containing the gene fragment encoding for i) the TDoT-domain, ii) a 3xGGGS linker (L) and iii) the enzyme *Ec*LDCc. The *Ec*LDCc gen was cloned into the vector by restriction with NdeI and NheI cleavage sites and ligation to attain a construct encoding a C-terminal fusion of the target enzyme to linker and TDoT. Oligonucleotide primer sequences used to amplify *Ec*LDCc gen are shown in the following Table S1. The final plasmid was sequenced by LGC genomics (Berlin, Germany).

Table S1: Primer sequences for amplification of *Ec*LDCc gen with NdeI and NheI cleavage sites (red)

name	sequence
NdeI_LDC_fw	5'- ATATATCATATGATGAACATCATCGCTATCATGGGCCC-3'
LDC_NheI_rv	5'- ATATATGCTAGCGCCTGCCATCTTAAGGACG-3'

Expression vector

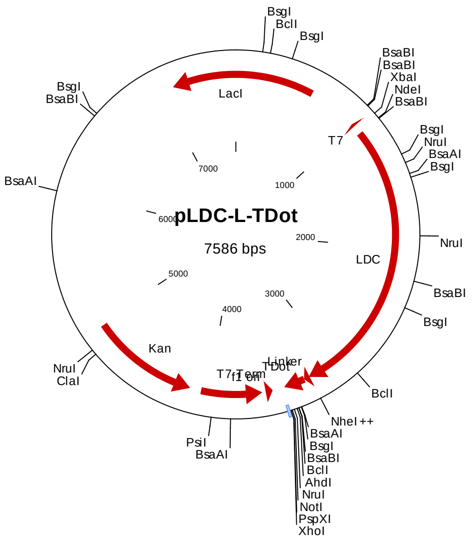

Figure S1: **pET28a vector containing the gene fusion encoding for *Ec*LDCc-L-TDoT. The DNA sequence of the vector is shown below.**

DNA-sequence of the pET28a vector containing the gene fusion encoding for *Ec*LDCc-L-TDoT

vector DNA (grey), start and stop codon of the *ldcc*-ORF (red), LDCc gen (black), linker (green), TDoT (orange), restrictions sites (blue)

CAGCCAGACGCAGACGCGCCGAGACAGAACTTAATGGGCCCGCTAACAGCGCGATTTGCT
GGTGACCCAATGCGACCAGATGCTCCACGCCCAGTCGCGTACCGTCTTCATGGGAGAAAA
TAATACTGTTGATGGGTGTCTGGTCAGAGACATCAAGAAATAACGCCGGAACATTAGTGC
AGGCAGCTTCCACAGCAATGGCATCCTGGTCATCCAGCGGATAGTTAATGATCAGCCCAC
TGACGCGTTGCGCGAGAAGATTGTGCACCGCCGCTTTACAGGCTTCGACGCCGCTTCGTT

CTACCATCGACACCACCACGCTGGCACCCAGTTGATCGGCGCGAGATTTAATCGCCGCGA
CAATTTGCGACGGCGCGTGCAGGGCCAGACTGGAGGTGGCAACGCCAATCAGCAACGACT
GTTTGCCCGCCAGTTGTTGTGCCACGCGGTTGGGAATGTAATTCAGCTCCGCCATCGCCG
CTTCCACTTTTTCCCGCGTTTTCGCAGAAACGTGGCTGGCCTGGTTCACCACGCGGGAAA
CGGTCTGATAAGAGACACCGGCATACTCTGCGACATCGTATAACGTTACTGGTTTCACAT
TCACCACCCTGAATTGACTCTCTTCCGGGCGCTATCATGCCATACCGCGAAAGGTTTTGC
GCCATTCGATGGTGTCCGGGATCTCGACGCTCTCCCTTATGCGACTCCTGCATTAGGAAG
CAGCCCAGTAGTAGGTTGAGGCCGTTGAGCACCGCCGCCGCAAGGAATGGTGCATGCAAG
GAGATGGCGCCCAACAGTCCCCCGGCCACGGGGCCTGCCACCATACCCACGCCGAAACAA
GCGCTCATGAGCCCGAAGTGGCGAGCCCGATCTTCCCCATCGGTGATGTCGGCGATATAG
GCGCCAGCAACCGCACCTGTGGCGCCGGTGATGCCGGCCACGATGCGTCCGGCGTAGAGG
ATCGAGATCTCGATCCCGCGAAATTAATACGACTCACTATAGGGGAATTGTGAGCGGATA
ACAATTCCCCTCTAGAAATAATTTTGTTTAACTTTAAGAAGGAGATATACATATGATGAA
CATCATCGCTATCATGGGCCCTCACGGTGTTTTCTACAAGGATGAGCCAATCAAGGAGCT
GGAATCCGCACTAGTTGCACAGGGCTTTCAGATCATCTGGCCCCAGAACTCCGTTGACCT
TCTCAAATTCATCGAGCACAATCCTCGCATTTGTGGTGTGATTTTTGACTGGGACGAGTA
CTCTCTTGATTTATGCTCCGACATCAACCAGCTCAACGAGTACCTGCCACTCTACGCATT
CATCAACACTCACTCCACCATGGACGTTTCCGTGCAGGACATGCGTATGGCACTCTGGTT
CTTTGAATACGCTCTGGGCCAGGCTGAGGACATCGCGATCCGCATGCGTCAGTACACCGA
CGAGTACCTGGACAACATCACCCCTCCATTCACCAAGGCTCTCTTCACCTACGTAAAGGA
ACGCAAGTACACTTTCTGCACCCCAGGCCACATGGGCGGCACCGCCTACCAGAAGTCCCC
AGTCGGATGCCTCTTCTACGACTTCTTCGGCGGTAACACTCTTAAGGCAGATGTCTCCAT
TTCCGTCACCGAGTTGGGCTCTCTGCTGGACCACACCGGCCCTCACCTGGAGGCAGAAGA
GTACATCGCTCGTACCTTCGGTGCTGAACAGTCCTACATCGTCACCAACGGTACTTCCAC
CAGCAACAAGATCGTTGGTATGTACGCAGCTCCTTCTGGCTCCACCCTGTTGATCGACCG
CAACTGTCACAAGTCCCTCGCGCATCTTCTTATGATGAACGATGTGGTCCCTGTATGGCT
GAAGCCAACCCGTAACGCTCTGGGCATCCTTGGCGGTATCCCCCGTCGCGAGTTCACCCG
TGATTCCATCGAGGAAAAGGTTGCAGCCACTACCCAGGCACAGTGGCCTGTCCACGCTGT
CATTACCAACTCGACCTACGACGGCCTGCTCTACAACACCGATTGGATCAAGCAGACCCT
AGATGTTCCTTCCATTCACTTCGACAGCGCATGGGTTCCTTACACTCACTTCCACCCAAT
CTACCAGGGTAAGTCCGGAATGTCCGGCGAGCGTGTCGCTGGCAAGGTTATCTTCGAAAC
CCAATCAACCCACAAGATGCTGGCTGCTCTCTCCCAGGCTTCTCTGATCCACATCAAGGG
CGAGTACGACGAGGAAGCTTTCAACGAGGCTTTCATGATGCACACCACCACCTCCCCATC
CTACCCTATCGTCGCGTCCGTCGAGACTGCTGCCGCAATGCTTCGCGGTAACCCAGGTAA
GCGCCTCATCAACCGTTCCGTTGAGCGCGCTCTTCACTTCCGTAAGGAAGTGCAGCGCCT
GCGTGAGGAATCTGACGGTTGGTTCTTCGACATTTGGCAGCCACCTCAGGTTGATGAGGC
CGAGTGCTGGCCAGTTGCTCCAGGTGAACAGTGGCACGGATTCAACGATGCAGATGCTGA
CCACATGTTTTTGGACCCGGTCAAGGTCACCATTCTTACTCCTGGTATGGATGAGCAGGG
CAACATGTCTGAGGAGGGTATCCCAGCTGCTCTGGTTGCAAAGTTCCTCGACGAACGTGG
CATCGTTGTTGAGAAGACCGGACCATACAACCTGCTGTTCCTGTTCAGCATCGGCATCGA
CAAAACCAAGGCAATGGGTCTGCTGCGCGGCCTTACCGAGTTCAAGCGCTCCTACGACCT
GAACCTTCGCATCAAGAATATGCTGCCGGACCTGTACGCTGAAGATCCTGATTTCTACCG
CAACATGCGCATCCAGGACCTCGCACAGGGCATCCACAAGCTCATTCGCAAGCACGACCT
GCCAGGCCTTATGCTCCGTGCATTCGATACCCTCCCAGAGATGATCATGACCCCTCACCA
GGCTTGGCAGCGCCAGATCAAGGGCGAGGTGGAAACCATCGCACTGGAGCAGCTGGTTGG
TCGTGTCTCCGCCAACATGATCCTGCCATATCCACCTGGCGTTCCGCTGCTGATGCCAGG
CGAGATGCTCACCAAGGAGTCCCGCACCGTATTGGACTTCCTTCTCATGTTGTGCTCTGT
TGGCCAGCACTACCCAGGCTTCGAGACCGACATCCACGGCGCTAAGCAAGATGAAGACGG
CGTTTACCGCGTTCGCGTCCTTAAGATGGCAGGCGCTAGCGGCGGTGGGTCTGGAGGCGG
CTCAGGTGGTGGGTCGGGATCCATCATTAACGAAACTGCCGATGACATCGTTTATCGCCT
GACAGTCATTATCGATGATCGCTACGAATCGCTGAAAAACCTGATTACCTTACGTGCAGA
TCGCTTGGAGATGATCATCAATGACAATGTGTCCACCATTCTCGCGAGCATTTAAGCGGC
CGCACTCGAGCACCACCACCACCACCACTGAGATCCGGCTGCTAACAAAGCCCGAAAGGA
AGCTGAGTTGGCTGCTGCCACCGCTGAGCAATAACTAGCATAACCCCTTGGGGCCTCTAA
ACGGGTCTTGAGGGGTTTTTTGCTGAAAGGAGGAACTATATCCGGATTGGCGAATGGGAC
GCGCCCTGTAGCGGCGCATTAAGCGCGGCGGGTGTGGTGGTTACGCGCAGCGTGACCGCT
ACACTTGCCAGCGCCCTAGCGCCCGCTCCTTTCGCTTTCTTCCCTTCCTTTCTCGCCACG
TTCGCCGGCTTTCCCCGTCAAGCTCTAAATCGGGGGCTCCCTTTAGGGTTCCGATTTAGT
GCTTTACGGCACCTCGACCCCAAAAAACTTGATTAGGGTGATGGTTCACGTAGTGGGCCA
TCGCCCTGATAGACGGTTTTTCGCCCTTTGACGTTGGAGTCCACGTTCTTTAATAGTGGA
CTCTTGTTCCAAACTGGAACAACACTCAACCCTATCTCGGTCTATTCTTTTGATTTATAA
GGGATTTTGCCGATTTCGGCCTATTGGTTAAAAAATGAGCTGATTTAACAAAAATTTAAC

GCGAATTTTAACAAAATATTAACGTTTACAATTTCAGGTGGCACTTTTCGGGGAAATGTG
CGCGGAACCCCTATTTGTTTATTTTTCTAAATACATTCAAATATGTATCCGCTCATGAAT
TAATTCTTAGAAAAACTCATCGAGCATCAAATGAAACTGCAATTTATTCATATCAGGATT
ATCAATACCATATTTTTGAAAAAGCCGTTTCTGTAATGAAGGAGAAAACTCACCGAGGCA
GTTCCATAGGATGGCAAGATCCTGGTATCGGTCTGCGATTCCGACTCGTCCAACATCAAT
ACAACCTATTAATTTCCCCTCGTCAAAAATAAGGTTATCAAGTGAGAAATCACCATGAGT
GACGACTGAATCCGGTGAGAATGGCAAAAGTTTATGCATTTCTTTCCAGACTTGTTCAAC
AGGCCAGCCATTACGCTCGTCATCAAAATCACTCGCATCAACCAAACCGTTATTCATTCG
TGATTGCGCCTGAGCGAGACGAAATACGCGATCGCTGTTAAAAGGACAATTACAAACAGG
AATCGAATGCAACCGGCGCAGGAACACTGCCAGCGCATCAACAATATTTTCACCTGAATC
AGGATATTCTTCTAATACCTGGAATGCTGTTTTCCCGGGGATCGCAGTGGTGAGTAACCA
TGCATCATCAGGAGTACGGATAAAATGCTTGATGGTCGGAAGAGGCATAAATTCCGTCAG
CCAGTTTAGTCTGACCATCTCATCTGTAACATCATTGGCAACGCTACCTTTGCCATGTTT
CAGAAACAACTCTGGCGCATCGGGCTTCCCATACAATCGATAGATTGTCGCACCTGATTG
CCCGACATTATCGCGAGCCCATTTATACCCATATAAATCAGCATCCATGTTGGAATTTAA
TCGCGGCCTAGAGCAAGACGTTTCCCGTTGAATATGGCTCATAACACCCCTTGTATTACT
GTTTATGTAAGCAGACAGTTTTATTGTTCATGACCAAAATCCCTTAACGTGAGTTTTCGT
TCCACTGAGCGTCAGACCCCGTAGAAAAGATCAAAGGATCTTCTTGAGATCCTTTTTTTC
TGCGCGTAATCTGCTGCTTGCAAACAAAAAAACCACCGCTACCAGCGGTGGTTTGTTTGC
CGGATCAAGAGCTACCAACTCTTTTTCCGAAGGTAACTGGCTTCAGCAGAGCGCAGATAC
CAAATACTGTCCTTCTAGTGTAGCCGTAGTTAGGCCACCACTTCAAGAACTCTGTAGCAC
CGCCTACATACCTCGCTCTGCTAATCCTGTTACCAGTGGCTGCTGCCAGTGGCGATAAGT
CGTGTCTTACCGGGTTGGACTCAAGACGATAGTTACCGGATAAGGCGCAGCGGTCGGGCT
GAACGGGGGGTTCGTGCACACAGCCCAGCTTGGAGCGAACGACCTACACCGAACTGAGAT
ACCTACAGCGTGAGCTATGAGAAAGCGCCACGCTTCCCGAAGGGAGAAAGGCGGACAGGT
ATCCGGTAAGCGGCAGGGTCGGAACAGGAGAGCGCACGAGGGAGCTTCCAGGGGGAAACG
CCTGGTATCTTTATAGTCCTGTCGGGTTTCGCCACCTCTGACTTGAGCGTCGATTTTTGT
GATGCTCGTCAGGGGGGCGGAGCCTATGGAAAAACGCCAGCAACGCGGCCTTTTTACGGT
TCCTGGCCTTTTGCTGGCCTTTTGCTCACATGTTCTTTCCTGCGTTATCCCCTGATTCTG
TGGATAACCGTATTACCGCCTTTGAGTGAGCTGATACCGCTCGCCGCAGCCGAACGACCG
AGCGCAGCGAGTCAGTGAGCGAGGAAGCGGAAGAGCGCCTGATGCGGTATTTTCTCCTTA
CGCATCTGTGCGGTATTTCACACCGCATATATGGTGCACTCTCAGTACAATCTGCTCTGA
TGCCGCATAGTTAAGCCAGTATACACTCCGCTATCGCTACGTGACTGGGTCATGGCTGCG
CCCCGACACCCGCCAACACCCGCTGACGCGCCCTGACGGGCTTGTCTGCTCCCGGCATCC
GCTTACAGACAAGCTGTGACCGTCTCCGGGAGCTGCATGTGTCAGAGGTTTTCACCGTCA
TCACCGAAACGCGCGAGGCAGCTGCGGTAAAGCTCATCAGCGTGGTCGTGAAGCGATTCA
CAGATGTCTGCCTGTTCATCCGCGTCCAGCTCGTTGAGTTTCTCCAGAAGCGTTAATGTC
TGGCTTCTGATAAAGCGGGCCATGTTAAGGGCGGTTTTTTCCTGTTTGGTCACTGATGCC
TCCGTGTAAGGGGGATTTCTGTTCATGGGGGTAATGATACCGATGAAACGAGAGAGGATG
CTCACGATACGGGTTACTGATGATGAACATGCCCGGTTACTGGAACGTTGTGAGGGTAAA
CAACTGGCGGTATGGATGCGGCGGGACCAGAGAAAAATCACTCAGGGTCAATGCCAGCGC
TTCGTTAATACAGATGTAGGTGTTCCACAGGGTAGCCAGCAGCATCCTGCGATGCAGATC
CGGAACATAATGGTGCAGGGCGCTGACTTCCGCGTTTCCAGACTTTACGAAACACGGAAA
CCGAAGACCATTCATGTTGTTGCTCAGGTCGCAGACGTTTTGCAGCAGCAGTCGCTTCAC
GTTCGCTCGCGTATCGGTGATTCATTCTGCTAACCAGTAAGGCAACCCCGCCAGCCTAGC
CGGGTCCTCAACGACAGGAGCACGATCATGCGCACCCGTGGGGCCGCCATGCCGGCGATA
ATGGCCTGCTTCTCGCCGAAACGTTTGGTGGCGGGACCAGTGACGAAGGCTTGAGCGAGG
GCGTGCAAGATTCCGAATACCGCAAGCGACAGGCCGATCATCGTCGCGCTCCAGCGAAAG
CGGTCCTCGCCGAAAATGACCCAGAGCGCTGCCGGCACCTGTCCTACGAGTTGCATGATA
AAGAAGACAGTCATAAGTGCGGCGACGATAGTCATGCCCCGCGCCCACCGGAAGGAGCTG
ACTGGGTTGAAGGCTCTCAAGGGCATCGGTCGAGATCCCGGTGCCTAATGAGTGAGCTAA
CTTACATTAATTGCGTTGCGCTCACTGCCCGCTTTCCAGTCGGGAAACCTGTCGTGCCAG
CTGCATTAATGAATCGGCCAACGCGCGGGGAGAGGCGGTTTGCGTATTGGGCGCCAGGGT
GGTTTTTCTTTTCACCAGTGAGACGGGCAACAGCTGATTGCCCTTCACCGCCTGGCCCTG
AGAGAGTTGCAGCAAGCGGTCCACGCTGGTTTGCCCCAGCAGGCGAAAATCCTGTTTGAT
GGTGGTTAACGGCGGGATATAACATGAGCTGTCTTCGGTATCGTCGTATCCCACTACCGA
GATATCCGCACCAACGCGCAGCCCGGACTCGGTAATGGCGCGCATTGCGCCCAGCGCCAT
CTGATCGTTGGCAACCAGCATCGCAGTGGGAACGATGCCCTCATTCAGCATTTGCATGGT
TTGTTGAAAACCGGACATGGCACTCCAGTCGCCTTCCCGTTCCGCTATCGGCTGAATTTG
ATTGCGAGTGAGATATTTATGCCAGC

Amino acid sequence of *Ec*LDCc-TDot

Ec**LDCc gen (black), linker (green), TDoT (orange), restrictions sites (blue)**

```
M N I I A I M G P H G V F Y K D E P I K E L E S A L V A Q G F Q I I W P Q N
S V D L L K F I E H N P R I C G V I F D W D E Y S L D L C S D I N Q L N E Y
L P L Y A F I N T H S T M D V S V Q D M R M A L W F F E Y A L G Q A E D I A
I R M R Q Y T D E Y L D N I T P P F T K A L F T Y V K E R K Y T F C T P G H
M G G T A Y Q K S P V G C L F Y D F F G G N T L K A D V S I S V T E L G S L
L D H T G P H L E A E E Y I A R T F G A E Q S Y I V T N G T S T S N K I V G
M Y A A P S G S T L L I D R N C H K S L A H L L M M N D V V P V W L K P T R
N A L G I L G G I P R R E F T R D S I E E K V A A T T Q A Q W P V H A V I T
N S T Y D G L L Y N T D W I K Q T L D V P S I H F D S A W V P Y T H F H P I
Y Q G K S G M S G E R V A G K V I F E T Q S T H K M L A A L S Q A S L I H I
K G E Y D E E A F N E A F M M H T T T S P S Y P I V A S V E T A A A M L R G
N P G K R L I N R S V E R A L H F R K E V Q R L R E E S D G W F F D I W Q P
P Q V D E A E C W P V A P G E Q W H G F N D A D A D H M F L D P V K V T I L
T P G M D E Q G N M S E E G I P A A L V A K F L D E R G I V V E K T G P Y N
L L F L F S I G I D K T K A M G L L R G L T E F K R S Y D L N L R I K N M L
P D L Y A E D P D F Y R N M R I Q D L A Q G I H K L I R K H D L P G L M L R
A F D T L P E M I M T P H Q A W Q R Q I K G E V E T I A L E Q L V G R V S A
N M I L P Y P P G V P L L M P G E M L T K E S R T V L D F L L M L C S V G Q
H Y P G F E T D I H G A K Q D E D G V Y R V R V L K M A G A S G G G S G G G
S G G G S G S I I N E T A D D I V Y R L T V I I D D R Y E S L K N L I T L R
A D R L E M I I N D N V S T I L A S I
```

Live cell imaging

After cultivation of *E. coli* BL21(DE3) containing *Ec*LDCc-CatIBs, 1 ml of the cell suspension in stationary growth phase was harvested by centrifugation at 15,800 x*g* for 2 min. The cell pellet was frozen at -80°C overnight and suspended in cell lysis buffer (50 mM sodium phosphate buffer, 100 mM NaCl, pH 8) to an OD_{600} of approx. 10. The cell suspension was transferred by a syringe to an in-house developed microfluidic chip, which was prepared for single-cell analysis and cultivation[2,3]. This device comprises hundreds of cultivation chambers for monolayer growth to enable imaging on a plane layer. Images were recorded with a phase contrast inverted epifluorescence microscope (TI-Eclipse, Nikon GmbH, Düsseldorf, Germany), additionally equipped with a CCD camera (Clara DR-3041, Andor Technology Plc., Belfast, UK), an LED light source (pE-100 white, CoolLed, Andover, UK), a Nikon Plan Apo 100 Ph3 DM Oil objective, and a Nikon Perfect Focus System for thermal drift compensation. Final images were processed by ImageJ (Wayne Rasband, USA).

Bioreactor cultivation of *C. glutamicum*

Bioreactor cultivations of the L-lysine producing *C. glutamicum* DM1945[4] were carried out in a 1 L scale using CGXII minimal medium[5]. For cultivation 1.5 L DASGIP vessels in a DASGIP Bioblock system (DASGIP GmbH, Jülich, Germany) were used as parallel cultivation system. Each bioreactor was equipped with an optical pO_2 sensor (Hamilton, Visiferm DO 225) and a pH electrode (Mettler-Toledo, 405-DPAS-SC-K8S/225/120). The

Bioblock controlling platform (DASGIP, DGCS4) was equipped with a monitoring system for pH and pO_2 (DASGIP, PH4PO4), an exhaust gas analyser (DASGIP, GA4), a pumping device for titration (DASGIP, MP8), a gassing system (DASGIP, MF4) and a system for temperature and agitation control (DASGIP, TC4SC4). During cultivation pO_2 was maintained to 30% by controlling the agitation rate (maximum 1200 min^{-1}) and a constant aeration rate of 1 volume air per volume fermentation broth per minute (vvm). The pH of the medium was maintained at pH 7 by the addition of 30% (v/v) aqueous H_3PO_4 and 17.7% (v/v) aqueous NH_3 solution. The temperature during cultivation was set to 30 °C.

Cultivations were directly inoculated from cryo-culture stocks to an initial $OD_{600\ nm}$ = 0.05. Cryo-culture stocks were prepared as described by Unthan *et al.* [6], except that culture medium CGXII containing 20% (v/v) glycerol was used for storage instead of 0.9% (w/v) NaCl. The cell suspension was harvested 6 h after the pO_2 returned to the initial value of 100% saturation. Subsequently, the cell fraction was separated from the liquid culture supernatant by centrifugation (20 min, 12,227 x*g*, 4 ºC) and then stored at -20 ºC.

Optimisation of the reaction system of *Ec*LDCc-CatIBs for the production of DAP from L-lysine

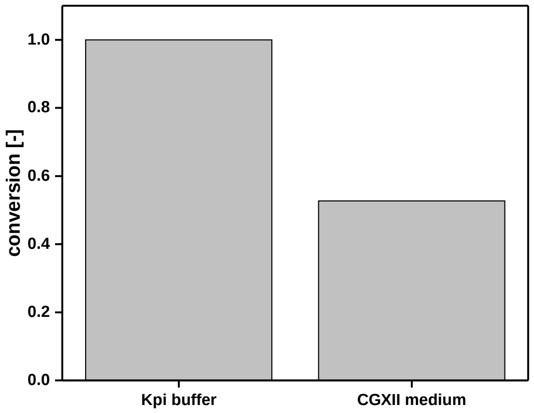

<u>Supplementary Figure S2:</u> Decarboxylation reaction by 1 mg ml^{-1} *Ec*LDCc-CatIBs with assay conditions: 10 mM L-lysine, 0.1 mM PLP, KPi buffer (50 mM, pH 7.5) or CGXII medium (pH 7.5, 20 g l^{-1} Glucose, 5 g l^{-1} urea) for 1 h at 30°C and 1000 rpm; the reaction was stopped by heating at 90°C for 2 min and subsequent centrifugation; Analysis was performed by HPLC analysis (see Methods in the main paper).

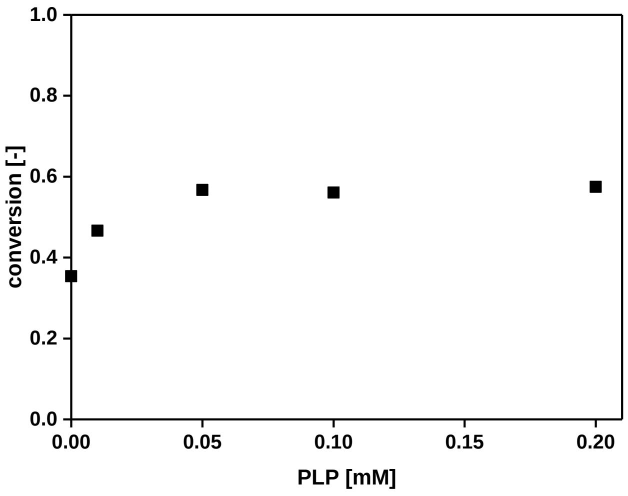

<u>Supplementary Figure S3:</u> Effect of different PLP concentrations (0.0, 0.01, 0.05, 0.1 and 0.2 mM) on the conversion of L-lysine to DAP by 0.5 mg ml^{-1} *Ec*LDCc-CatIBs; experimental conditions: 10 mM L-lysine, KPi buffer (50 mM, pH 8, supplemented with PLP as indicated) for 30 min at 30°C and 1000 rpm; the reaction was stopped by heating at 90°C for 2 min and subsequent centrifugation; Analysis was performed by HPLC analysis (see Methods in the main paper), n = 1.

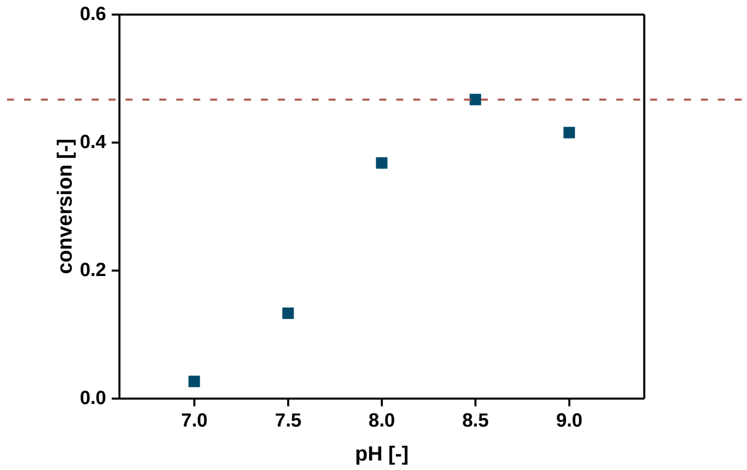

<u>Supplementary Figure S4:</u> Determination of the optimal pH for the *Ec*LDCc-CatIB-catalysed decarboxylation of L-lysine. Experimental conditions: 0.5 mg ml^{-1} lyophilized *Ec*LDCc-CatIBs, 10 mM L-lysine, 0.1 mM PLP, CGXII medium (pH 7, 7.5, 8, 8.5, 9) for 30 min at 30°C and 1000 rpm; the reaction was stopped by 1:5 dilution with methanol and subsequent centrifugation; Analysis was performed by HPLC analysis (see Methods in the main paper), n=3

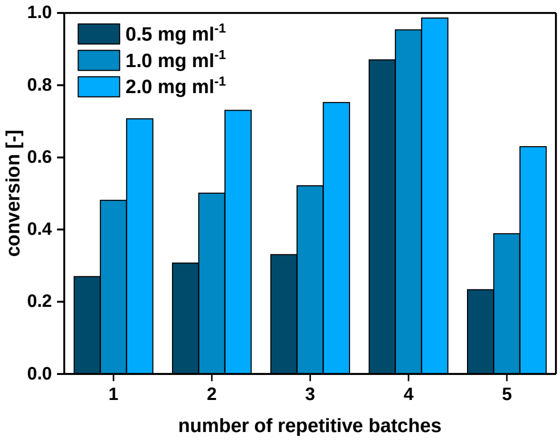

<u>Supplementary Figure S5:</u> Repetitive batch reactions for DAP production catalysed by different concentrations of *Ec*LDCc-CatIBs (0.5, 1 or 2 mg ml^{-1}); Experimental conditions: total volume 1 ml, 100 mM L-lysine, 0.01 mM PLP in cell free culture supernatant (CGXII medium, pH 8) for 2 h at 30°C and 1000 rpm, except batch 4: 16 h overnight. The CatIBs used in the first batch were reused in the next batch after centrifugation and resuspension in a fresh reaction solution. The reaction was stopped by 1:5 dilution with methanol and subsequent centrifugation; HPLC analysis (see Methods in the main paper).

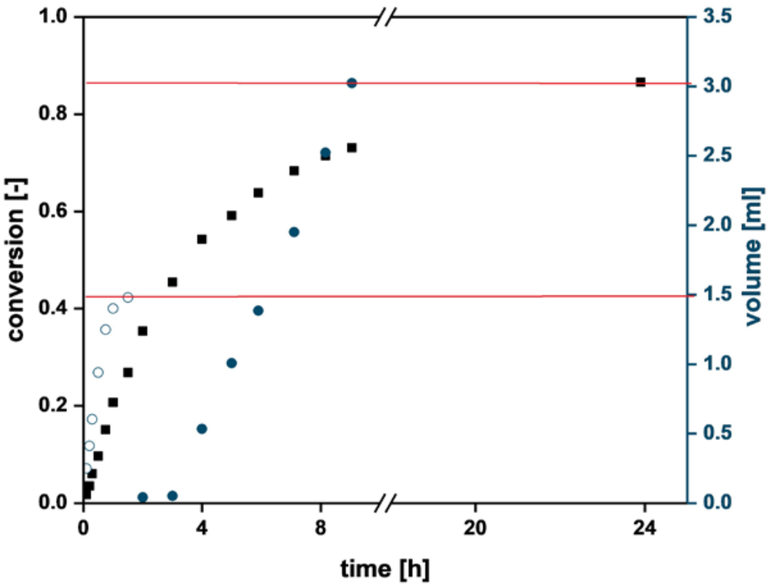

<u>Supplementary Fig. S6:</u> Shows the conversion of 1 M L-lysine in a 30 ml batch reactor (cf. Fig. 7, main paper) supplemented with information on the titration volumes of 2 N NaOH (open circles) and 5% HCl aq. (closed circles). As the reaction is performed in "used" CGXII-medium, the initially included phosphate buffer was metabolised by the growing *C. glutamicum* lysine producer strain. Thus the buffer capacity of the medium was low. The decarboxylation of lysine yields CO_2 and DAP in equimolar concentration. The formation of CO_2 is assumed to decrease pH, which requires titration with NaOH in the beginning of the reaction. With progressive conversion the increasing concentration of DAP led to an increase of pH, which was compensated by addition of HCl.

Control experiments with soluble LDCc in *E. coli*

Preparation of the whole cell biocatalyst: *Ec*LDCc was produced in *E. coli* BL21(DE3) as described in Methods in the main paper. Afterwards the cell pellet was frozen overnight at -20°C and a 10 % (w/v) suspension in MilliQ water was prepared for lyophilisation (Christ ALPHA 1-3 LD Plus, Martin Christ Gefriertrocknungsanlagen GmbH, Osterode, Germany). The dried pellet was weighted and stored at -20°C for further use.

Production of DAP using whole cells with soluble LDCc and *Ec*LDCc-CatIBs: Experimental conditions: lyophilised *E. coli* BL21(DE3) cells containing LDCc (0,01 – 1 mg/ml) or *Ec*LDCc-CatIBs (0.5 mg/ml) were suspended in reaction buffer containing 10 mM L-lysine, 50 mM KPi, pH 8, 0.1 mM PLP, respectively.

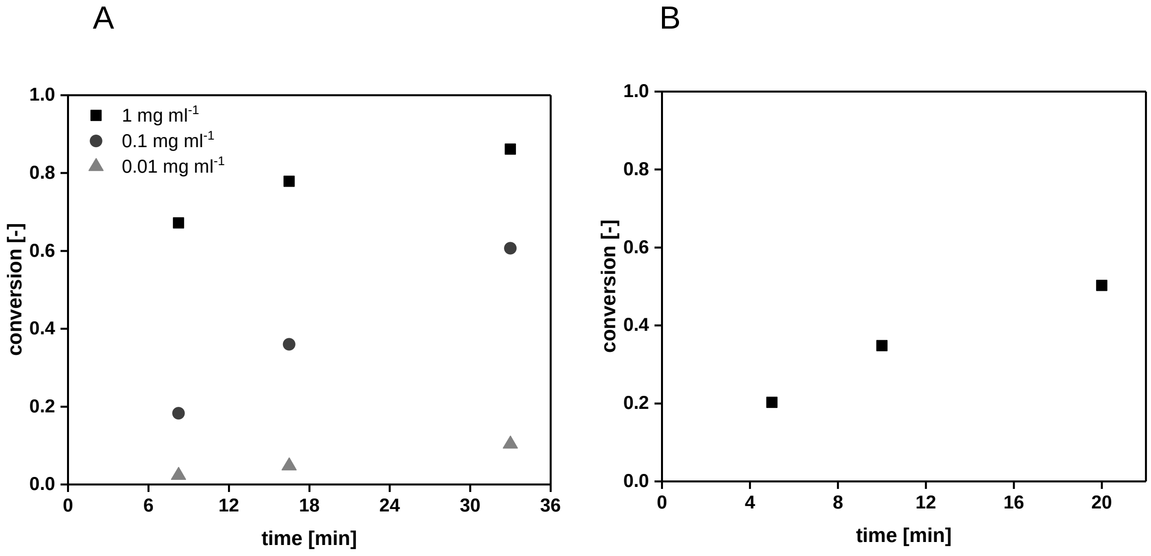

Supplementary Fig. S7: A: shows results obtained with the whole cell biocatalyst containing soluble LDCc in 3 different concentrations. B: shows results obtained with 0.5 mg/ml *Ec*LDCc-CatIBs.

These studies demonstrate that with 0.1 mg/ml of these whole cell biocatalyst suspended in KPi-buffer 10 mM L-Lys are converted to 50 % in approximately 25 min. The same conversion is achieved with 0.5 mg/ml CatIBs in 20 min. So the *Ec*LDCc-CatIBs perform equally well or even better than the whole cell biocatalyst.

HPLC calibration curves

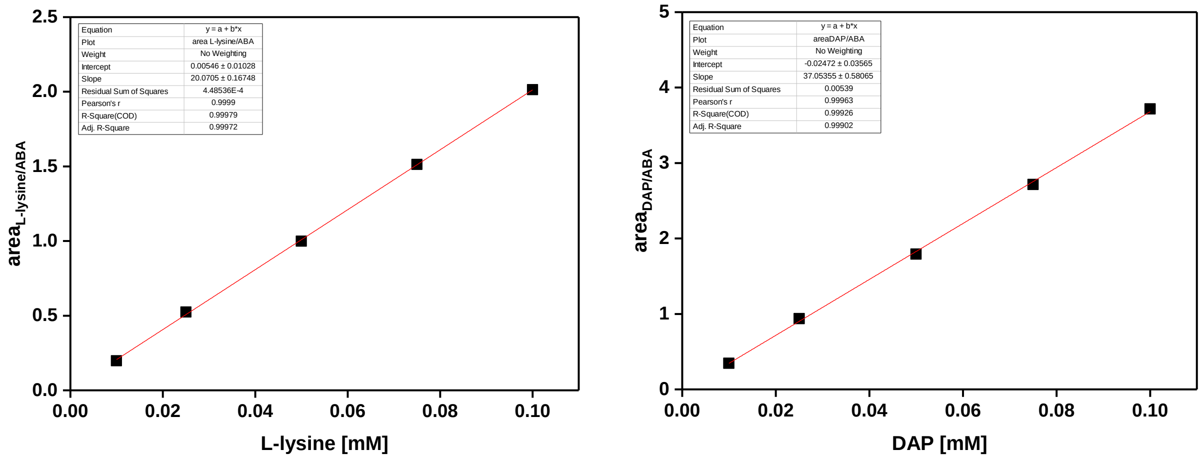

<u>Supplementary Figure S8:</u> Calibration curve for DAP and L-lysine with the internal standard ABA (alpha-aminobutyric acid), HPLC analysis (see Methods in the main paper).

References

1. Diener, M., Kopka, B., Pohl, M., Jaeger, K.-E. & Krauss, U. Fusion of a coiled-coil domain facilitates the high-level production of catalytically active enzyme inclusion bodies. *ChemCatChem* **8,** 142–152 (2016).
2. Grünberger, A. *et al.* Microfluidic picoliter bioreactor for microbial single-cell analysis: Fabrication, system setup, and operation. *J. Vis. Exp.* 1–11 (2013). doi:10.3791/50560
3. Grünberger, A. *et al.* Spatiotemporal microbial single-cell analysis using a high-throughput microfluidics cultivation platform. *Cytom. Part A* **87,** 1101–1115 (2015).
4. Limberg, M. H. *et al.* Metabolic profile of 1,5-diaminopentane producing *Corynebacterium glutamicum* under scale-down conditions: Blueprint for robustness to bioreactor inhomogeneities. *Biotechnol. Bioeng.* **114,** 560–575 (2017).
5. Keilhauer, C., Eggeling, L. & Sahm, H. Isoleucine synthesis in *Corynebacterium glutamicum*: molecular analysis of the ilvB-ilvN-ilvC operon. *J. Bacteriol.* **175,** 5595–603 (1993).
6. Unthan, S. *et al.* Beyond growth rate 0.6: What drives *Corynebacterium glutamicum* to higher growth rates in defined medium. *Biotechnol. Bioeng.* **111,** 359–371 (2014).
